# Efficient genome replication of hepatitis B virus using adenovirus vector: a compact pregenomic RNA-expression unit

**DOI:** 10.1038/srep41851

**Published:** 2017-02-03

**Authors:** Mariko Suzuki, Saki Kondo, Manabu Yamasaki, Norie Matsuda, Akio Nomoto, Tetsuro Suzuki, Izumu Saito, Yumi Kanegae

**Affiliations:** 1 Laboratory of Molecular Genetics, The Institute of Medical Science, The University of Tokyo, Tokyo, 108-8639, Japan; 2 Laboratory of Virology, Institute of Microbial Chemistry (BIKAKEN), Microbial Chemistry Research Foundation, Tokyo 141-0021, Japan; 3 Department of Virology and Parasitology, Hamamatsu University School of Medicine, Shizuoka 431-3192, Japan; 4 Core Research Facilities of Basic Science (Molecular Genetics), Research Center for Medical Science, Jikei University School of Medicine, Tokyo 105-8461, Japan.

## Abstract

The complicated replication mechanisms of hepatitis B virus (HBV) have impeded HBV studies and anti-HBV therapy development as well. Herein we report efficient genome replication of HBV applying adenovirus vectors (AdVs) showing high transduction efficiency. Even in primary hepatocytes derived from humanized mice the transduction efficiencies using AdVs were 450-fold higher compared than those using plasmids. By using an expression unit consisting of the CMV promoter, 1.03-copy HBV genome and foreign poly(A) signal, we successfully generated an improved AdV (HBV103-AdV) that efficiently provided 58 times more pregenomic RNA than previously reported AdVs. The HBV103-AdV-mediated HBV replication was easily and precisely detected using quantitative real-time PCR in primary hepatocytes as well as in HepG2 cells. Notably, when the AdV containing replication-defective HBV genome of 1.14 copy was transduced, we observed that HBV DNA-containing circular molecules (pseudo-ccc DNA) were produced, which were probably generated through homologous recombination. However, the replication-defective HBV103-AdV hardly yielded the pseudo-ccc, probably because the repeated sequences are vey short. Additionally, the efficacies of entecavir and lamivudine were quantitatively evaluated using this system at only 4 days postinfection with HBV103-AdVs. Therefore, this system offers high production of HBV genome replication and thus could become used widely.

Hepatitis B virus (HBV) is a causative pathogen of chronic liver disease, which increases the risk of development not only of liver cirrhosis but also of hepatocellular carcinoma. About 240 million people are chronic carriers of HBV and 686,000 people die due to cirrhosis and liver cancer secondary to HBV infection, every year^1^. Although alpha interferon (IFN) shows therapeutic effect for the majority of hepatitis C virus patients, approximately 50% of HBV patients acquire resistance to IFN^2^,^3^,^4^. Nucleos(t)ide analogues, such as lamivudine and entecavir, have shown significant efficacy in inhibiting reverse transcription in some patients with HBV, but the problem of resistant viruses has not yet been solved^5^. Moreover, clearance of HBV genome from chronic infection is unlikely using known drugs. Therefore, development of therapies for HBV infection with improved efficacy and safety is desired, and convenient and accurate assay of HBV genome replication is warranted. In addition, a simple and efficient detection method of HBV infection/genome replication will contribute not only to the development of anti-HBV drugs but also to the studies of the HBV replication mechanism.

The HBV virion contains an incomplete double-stranded DNA genome (relaxed circular: rc), which is then converted to a covalently closed circular (ccc) DNA in the nuclei during the virus life cycle. Since pregenomic (pg) RNA is transcribed from ccc DNA, the stable presence of ccc DNA genome in nuclei as a template establishes chronic HBV infection^6^. Thus, the complicated replication mechanisms of HBV have hindered HBV studies as well as HBV therapies. Furthermore, there are only few methods to examine HBV genome replication *in vitro* and *in vivo*, because HBV proliferates very poorly in these systems. Three methods are generally used: (i) Transfection of plasmids containing the 1.2-copy HBV genome is the most popular method to study HBV genome replication because of the ease of plasmid construction. However, plasmids are not necessarily introduced uniformly into cells, and so this method may not be suitable for the quantitative study of HBV genome replication e.g. high-throughput screening. Moreover, primary hepatocytes, for instance PXB cells^7^,^8^, can hardly be used for plasmid transfection; (ii) HBV-producing hepatoblastoma cell lines^9^,^10^,^11^,^12^, in which multiple HBV genomes are integrated, are essentially suitable for drug screening. Because the copy number of pg RNA and the replicating HBV DNA genome are almost constant in the cells. However, only few pg RNAs are generated from the HBV genome in these cell lines, and consequently it takes a long time for sufficient HBV-replicating genome to accumulate for detection; (iii) Applications of Dane particles to HBV studies have been limited, because the host range of HBV is very narrow. But since human sodium taurocholate cotransporting polypeptide (hNTCP) has been revealed as a receptor for HBV infection^13^,^14^, by using hNTCP-expressing cells, Dane particles can become applied to experiments of HBV. However, this method may not be suitable for mutagenesis analysis of HBV.

Using adenovirus vectors (AdVs) for the introduction of HBV genome into cells is one of the most convenient methods to study HBV genome replication. Its high transduction efficiency contributes to the production of replicating HBV genome in cells, there have been several reports of application of AdVs containing a 1.29-copy HBV genome^15^,^16^. However, in those reports, pg RNA was transcribed using endogenous core promoters, whose activities were insufficient for efficient genome replication. Moreover, a GFP expression unit using a strong promoter was inserted upstream of the HBV expression unit in the AdV genome, and thus the two independent promoters in the same cloning site of the AdV genome might interfere with each other. Therefore, those reported AdVs expressing HBV pg RNAs may not have been optimised for efficient replication of the HBV genome.

In this study, we have established a new system to facilitate the detection of the replicating HBV genome using AdVs, named “HBV103-AdV system”. In this system, 1.03-copy HBV genome, which is a compact HBV sequence for replication-competent HBV genome, was used together with CMV promoter^17^ and rabbit β-globin poly(A) signal^18^ to transcribe pg RNA. Advantages of the HBV103-AdV system are not only to efficiently supply pg RNA because of a strong promoter and efficient transduction, but also to suppress the production of pseudo-ccc DNA irrespective of HBV genome replication. Furthermore, we examined whether the HBV103-AdV system could be applied to various cells. Notably, since HBV genome is hardly transduced using plasmid transfection in these cells, the application of this system to human primary hepatocytes is probably valuable. In addition, as the HBV103-AdV system should be capable of detecting the replicating HBV genome faster than other methods and indicating refined dose response, it will be useful for high-throughput screening of new anti-HBV drugs.

## Results

### AdVs showed considerably higher transduction efficiencies than plasmid transfection

**Figure 1:**
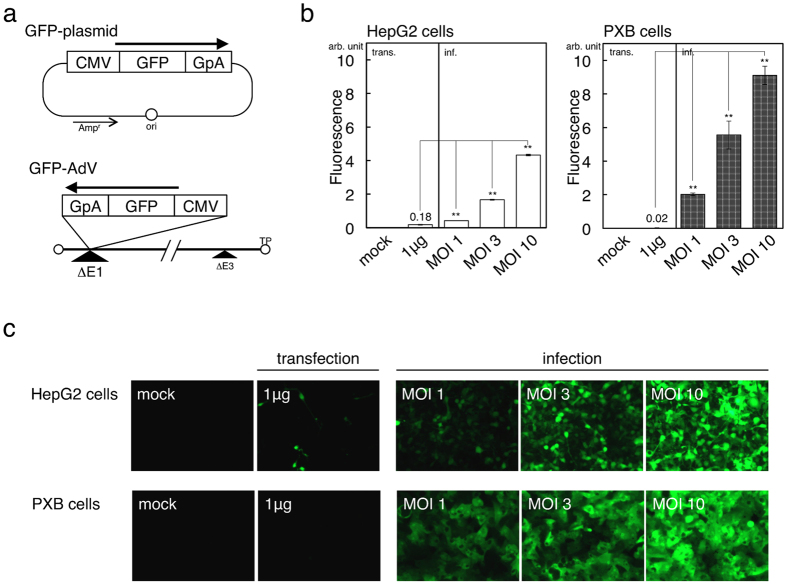
Transduction efficiencies of GFP using plasmids and AdVs. (**a**) Structures of GFP-expressing plasmids and AdVs. The sequential boxes represent the expression unit and arrows show the orientation of transcription. ‘CMV’, CMV promoter; ‘GpA’, rabbit β-globin poly(A) signal. (**b**) GFP fluorescence was quantified by Varioskan Flash. Cells were transfected with GFP-expressing plasmids or infected with GFP-expressing AdVs by the indicated amount and MOI. ‘arb. unit’, arbitrary unit. *n* = 3. (**c**) Images obtained using fluorescent microscopy in the same manner as that described in (**b**). Error bars represent ± s.d.; mock, mock infection of the indicated cells; **P < 0.01.

To compare the transduction efficiencies of AdV versus plasmid, we introduced GFP into HepG2 cells and PXB cell using either plasmid or AdV, and examined both the expression levels of GFP and the percentages of GFP-positive cells (Fig. 1b and c). Hepatoblastoma-derived HepG2 cells have generally been used in HBV studies to efficiently detect HBV genome replication. PXB cells, which are employed as human primary hepatocytes, not only retain normal hepatic function but also show tolerant to HBV infection, so have been an appropriate means for *in vitro* analysis of HBV proliferation. Whereas, the drawback of these cells is that transduction efficiency is normally low.

Both plasmids and AdVs contained the same expression unit (Fig. 1a). When 10 μg of plasmids were used for the transfection, HepG2 cells were damaged. Therefore, we adopted 3 μg for transfection experiments throughout (Supplementary Fig. S1). Only 5% of HepG2 cells expressed GFP following plasmid transfection, whereas AdVs were able to introduce GFP into almost all HepG2 cells at a multiplicity of infection (MOI) of 10. Indeed, the AdVs infecting HepG2 cells at MOI 10 showed a 24-fold higher fluorescence than that achieved with plasmid transfection.

In PXB cells, AdVs showed much higher transduction efficiencies and expression levels than plasmids. Plasmids showed poor ability to introduce the GFP gene into PXB cells, while AdVs at MOI 10 showed more than 90% GFP-positive cells, and the ratio of positive cells remained 90% even at MOI 3. Furthermore, in PXB cells AdVs at MOI 10 produced 450-fold more GFP than plasmids. These results suggest that AdVs have the obvious advantage of high transduction efficiencies compared with plasmids, especially in PXB cells, which is valuable for HBV studies. Cytotoxicity was not observed in PXB cells infected with AdVs at MOI 10. It should be noted that AdVs even at MOI 1 demonstrated uniform GFP expression, but plasmids expressed GFP only in limited number of cells. Additionally in Huh-7 cells, fluorescence, representing GFP-expressing levels, in the transfected cells was higher than in the cells at MOI 10 (Supplementary Fig. S2). However, some cells showed strongly fluorescent in the panel of the transfected cells, and GFP-positive cells were evenly observed in the whole field of vision in the panel of MOI 10. Thus, the application of AdVs makes it easy to regulate the amount of transgene transduced into the cells.

### The HBV pg RNA was abundantly produced using CMV promoter and rabbit β-globin poly(A) signal on the AdV genome

**Figure 2:**
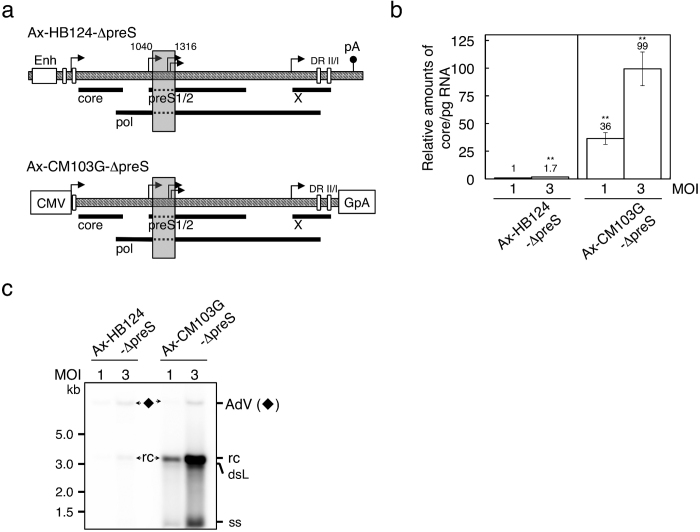
Expression levels of pg RNA of HBV in HepG2 cells infected with AdVs. (**a**) Schematic representation of the pg RNA expression unit on the AdVs. Ax-HB124-ΔpreS transcribes mutant HBV pg RNAs under the control of the endogenous core promoter. Ax-CM103G-ΔpreS produces pg RNAs using CMV promoter. The deleted region of the mutant HBV genome ‘ΔpreS’ is shown as a grey box. Bold and broken lines represent the ORFs of HBV proteins and deleted regions, respectively. Right-angled arrow, transcription initiation site; Enh, enhancer region of endogenous core promoter; pA, native polyadenylation signal of HBV genome; DR II/I, direct repeat sequences of HBV genome. The other representations are the same as in Fig. 1. (**b**) Relative expression levels of HBV RNAs. HepG2 cells were infected with Ax-HB124-ΔpreS and Ax-CM103G-ΔpreS at MOI 1 and 3. Quantities of core/pg RNA were determined by qPCR. Amount of core/pg RNA in cells infected with Ax-HB124-ΔpreS at MOI 1 is regarded as 1. *n* = 3. Error bars represent ± s.d.; **P < 0.01. (**c**) Detection of replicating HBV genome. Total DNA from either infected HepG2 cells with Ax-HB124-ΔpreS or Ax-CM103G-ΔpreS were analysed using Southern blotting. AdV (◆), *Kpn*I-digested genome of adenovirus vectors; rc, relaxed circular DNA genome of HBV; dsL, double-stranded linear DNA genome of HBV; ss, single stranded DNA of HBV. Overexposure of blots and full-length blots are presented in Supplementary Figs S5 and S9, respectively.

To obtain high expression of pg RNA, we utilized CMV promoter and rabbit β-globin poly(A) signal, which is known to contribute to high mRNA-stability^19^,^20^. We constructed two AdVs: Ax-HB124-ΔpreS and Ax-CM103G-ΔpreS, which contain 1.24 copy and 1.03 copy HBV genomes, respectively (Fig. 2a). In the pg RNA, transcribed from the CM103G HBV genome, the original HBV poly(A) sequence is disrupted and replaced by the foreign poly(A) sequence (Supplementary Fig. S3b). The poly(A) sequences are subsequently removed at the step where the terminal-protein domain of pol attaches as a protein primer to the downstream DRI and the minus-strand DNA synthesis starts. The HBV genome ΔpreS used here lacks most of the preS1 and preS2, while maintaining the polymerase activity^21^ (deleted sequences are shown in Supplementary Fig. S4).

HepG2 cells were infected with either Ax-HB124-ΔpreS or Ax-CM103G-ΔpreS at MOI 1 and MOI 3. The amounts of mRNAs from infected cells at 3 days postinfection were then measured using quantitative real-time PCR (qPCR) with the primers shown in Supplementary table S1 were designed to detect both core and pg RNA, because they share the same promoter. The ratios of core/pg RNA level to the amount of core/pg RNA from Ax-HB124-ΔpreS-infected cells at MOI 1 (Fig. 2b) demonstrated that the activity of CMV promoter was much higher than that of the endogenous core promoter: Ax-CM103G-ΔpreS produced 36 times more core/pg RNA than Ax-HB124-ΔpreS at MOI 1. Similarly, at MOI 3, Ax-CM103G-ΔpreS demonstrated about 58-fold more transcription of core/pg RNA than Ax-HB124-ΔpreS.

Moreover, production of this large amount of pg RNA probably contributes to efficient HBV genome replication (Fig. 2c, Supplementary Fig. S5a). Total cellular DNA from the cells infected with the indicated AdVs was digested by *Kpn*I, which cuts AdV and cellular genome, but not HBV genome. Bands representing rc DNA genome from Ax-CM103G-ΔpreS-infected cells were strongly detected compared with Ax-HB124-ΔpreS, while bands representing AdV genomic DNA (◆) were observed at similar levels. Therefore, CMV promoter-driven expression of the HBV genome may offer a new efficient detection system.

### Pseudo-ccc DNA was generated from the repeated sequences of HBV

Because the endogenous core promoter overlaps the 3′ end of the X open reading frame (ORF) and the 5′ end of the preCore/Core ORF, 1.29-copy HBV genome overlap by 920 bp is necessary for HBV gene expression in all the previously reported AdVs^15^,^16^. However, it is possible that large repeated sequences may cause homologous recombination during vector production. Because of homologous recombination, the excised circular DNA of 1.29 copy HBV genome must be generated as pseudo-ccc DNA molecules identical to ccc DNA, which should be detected together with ccc DNA in PCR, qPCR and Southern analysis.

**Figure 3:**
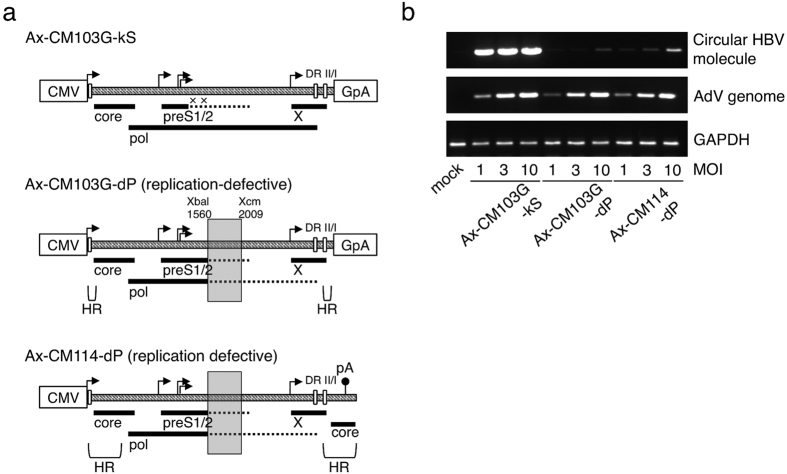
Detection of pseudo-ccc DNA. (**a**) Schematic representation of the HBV expression unit on the AdVs. Ax-CM103G-kS harbours a replicate-competent mutant HBV genome. Crosses represent silent mutation of HBV S protein. Deleted region of the mutant HBV genome “dP” on Ax-CM103G-dP and Ax-CM114-dP are shown as a grey box. Bold and broken lines represent the ORFs of HBV proteins and non-functional region, respectively. Right-angled arrow, transcription initiation site; DR II/I, direct repeat sequences of HBV genome; HR, homologous region. The other representations are the same as in Figs 1 and 2. (**b**) PCR detection of the circular HBV genome, AdV genome and GAPDH. HepG2 cells were infected with the indicated AdVs. Mock, mock infection of HepG2 cells. Full-length blots are presented in Supplementary Fig. S9.

Ax-CM103G-kS and Ax-CM103G-dP possess the extremely shorter overlapping HBV genome, while Ax-CM114-dP, which contains a 1.14 copy of HBV genome, retaining the HBV native poly(A) signal: overlapping length of Ax-CM103G-kS and Ax-CM103G-dP was only 102 bp, and that of Ax-CM114-dP was 376 bp (Fig. 3a). The mutant HBV genome termed kS is a replication-competent HBV genome that lacks the production of the small S protein (Supplementary Fig. S6) because of point mutations at two possible initiation codons (Supplementary Fig. S4), maintaining the intact pol coding frame. The mutant HBV genome, termed dP, is replication-defective, which lacks a part of the essential polymerase domain (Supplementary Fig. S4).

To examine whether the short overlapping region actually resulted in successful suppression of the homologous recombination of HBV genomes, circular HBV DNA molecules were examined to detect using semi-quantitative PCR (Fig. 3b). Total DNA was prepared from HepG2 cells infected with the above three AdVs at the indicated MOIs to compare the amounts of circular DNA molecules from Ax-CM103G-dP with those from Ax-CM114-dP. The PCR primers were designed to specifically detect circular DNA molecules, but not linear HBV DNA inserted into AdV genomes, because they are prepared tail to tail on the linear HBV DNA (Supplementary Fig. S7). Remarkably, the circular DNA molecules from Ax-CM114-dP were distinctly detected at MOI 10, although the mutant HBV genome dP must not be able to replicate by itself. In contrast, those from Ax-CM103G-dP were hardly detected. Replicating HBV genomes from Ax-CM103-kS were clearly detected. These results suggested that the application of CMV promoter and foreign poly(A) to HBV expression on AdVs, i.e. the HBV103-AdV system, could avoid the generation of “pseudo ccc DNA” resulting from the homologous recombination.

### Replicating HBV genome was strongly detected in HepG2 and PXB cells using the HBV103-AdV system

To estimate the efficiencies of HBV103-AdV system-mediated HBV genome replication, replicating HBV genomes were detected using Southern analysis and qPCR. The AdVs harboring either the mutant HBV genome kS (Ax-CM103G-kS) or the replication-defective HBV genome dP (Ax-CM103G-dP) were compared with plasmids containing the same HBV expression unit (pCM103G-kS or pCM103G-dP), with regards to HBV genome replication.

**Figure 4:**
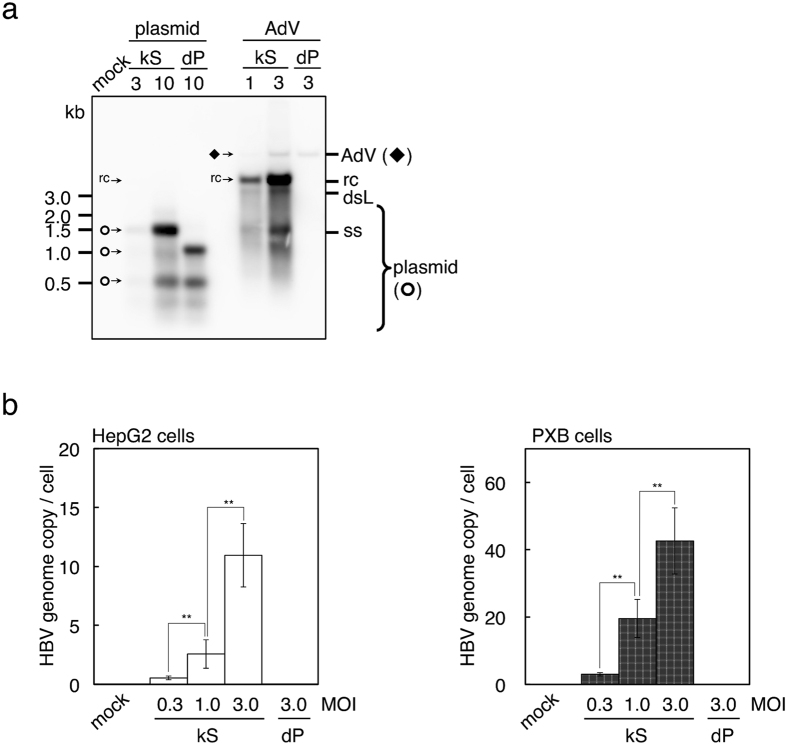
Detection and quantification of replicating HBV genome. Cells were infected with Ax-CM103G-kS (kS) or Ax-CM103G-dP (dP) by the indicated MOIs, or transfected with plasmids possessing the same mutant HBV expression units. (**a**) Detection of the replicating HBV genome. Total DNA either from infected or transfected HepG2 cells were analysed using Southern blot analysis. AdV (◆), *Kpn*I-digested genome of adenovirus vector; rc, relaxed circular DNA genome of HBV; dsL, double-stranded linear DNA genome of HBV; ss, single stranded DNA of HBV; plasmid (**¢**), *Hind*III and *Dpn*I-digested plasmid fragments; mock, mock infection. Overexposure of blots and full-length blots are presented in Supplementary Figs S5 and S9, respectively. (**b**) Replicating HBV genomes were quantified using qPCR. Total DNA from infected HepG2 or PXB cells were used to performed qPCR. *n* = 3. Error bars represent ± s.d.; mock, mock infection of the indicated cells; **P < 0.01.

As a result of Southern blot analysis, two bands, representing rc DNA genome and double-stranded DNA genome of HBV, were strongly detected following infection with the AdVs (Fig. 4a, Supplementary Fig. S5b). Therefore, HBV genome replication mediated by the HBV103-AdV system should be much more efficient and more readily detectable compared with plasmid transfection. It should be noted that total cellular DNA was used in these experiments to detect replicating HBV genomes, without any concentration step.

Next, we attempted to quantitatively determine HBV genome replication (Fig. 4b). Total cellular DNA was extracted from cells infected with the AdVs at the indicated MOI. Quantitative PCR was performed to detect rc DNA and ccc DNA of the HBV genome. Ax-CM103G-kS at MOI 3 in HepG2 cells produced approximately 10 copies of HBV genome per cell. Furthermore, PXB cells infected with Ax-CM103G-kS at MOI 3 showed much higher efficiencies of HBV replication than HepG2 cells: more than 40 copies of the replicating HBV genome were detected in individual cells. Additionally, production of the replicating HBV genome may depend on the infectious dose of AdVs. These results suggested that the AdVs represent a superior means of producing replicating HBV genome compared with the plasmids.

### The AdV103-HBV system is able to estimate inhibitory potency of anti-HBV drugs

To evaluate the usefulness of the HBV103-AdV system for antiviral testing, we investigated the effects of the reverse transcriptase inhibitors currently used for HBV therapy, entecavir and lamivudine, on virus DNA synthesis in Huh-7 cells infected with Ax-CM103G-ΔpreS. To allow rapid drug screening in future studies, we optimised the assay conditions in a 96-well format, and used the virus polymerase-deleted mutant, Ax-CM103G-dP, for background subtraction. The quality of our assay was evaluated by determining the Z’-factor^22^. This statistical parameter that exceeds 0.5 indicates the suitability of the assay for hit identification in a high-throughput screening program. In the assay conditions, coefficient of variation, signal-to-background ratio, and signal-to-noise ratio were 12.0 ± 0.8, 7668 ± 2892, and 16815 ± 9136, respectively, between plates from three independent experiments. The Z’ factor was 0.64 ± 0.02, indicating that our assay was accurate and robust enough to use in a drug screening.

**Figure 5:**
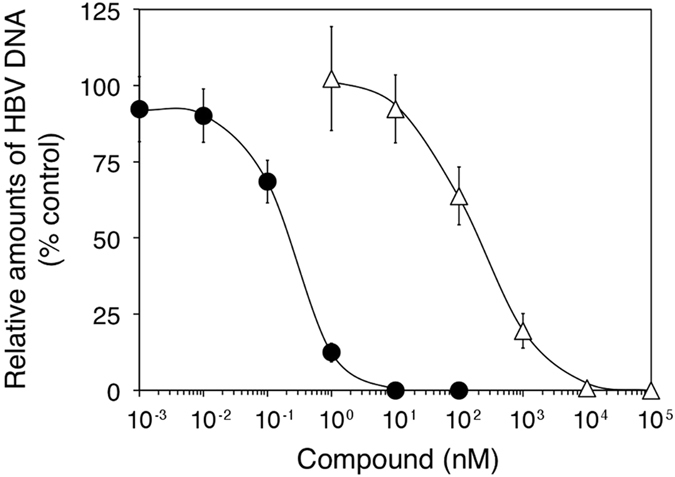
Inhibition of HBV genome replication in Huh-7 cells infected with HBV103-AdV by HBV reverse transcriptase inhibitors. Huh-7 cells were infected with Ax-CM103G-ΔpreS, and incubated with entecavir (closed circles) and lamivudine (open triangles). Replicating virus genomes in cells at 4 days postinfection were quantified by qPCR. Relative amounts of virus DNA are shown as a percentage of the untreated control (DMSO). *n* = 3. Error bars represent ± s.d.

As shown in Fig. 5, entecavir and lamivudine inhibited virus DNA accumulation in the cells in a dose-dependent manner, with a 50% effective concentration (EC_50_) of 0.2 and 189 nM, respectively. These values were comparable to those previously obtained by both duck HBV infection of primary duck hepatocytes^23^ and transfection of plasmid containing human HBV genome^24^,^25^. Besides, two drugs had little effects on the GFP fluorescence intensity when the cells were coinfected with HBV103-AdV and GFP-AdV, as well as the amount of adenoviral vector (Supplementary Fig. S8), confirming their specificity for HBV DNA synthesis process. These results indicated that our constructed HBV103-AdVs are a valuable tool to estimate the inhibitory potency of test compounds on the virus genome replication.

## Discussion

AdVs have advantages in transduction efficiency for various cell types. HBV genome replication mediated by AdVs possessing 1.29 copy HBV genome, which includes the endogenous core promoter and the native poly(A) signal, have already been carried out^15^,^16^. These conventional AdVs were used not only for investigations into the time-course of HBV proliferation but also for experiments using human primary hepatocytes derived from biopsy specimens of patients. Besides, some AdVs were also reported as vehicles for recombinant HBV genomes in which an arbitrary transgene was substituted for a part of HBV gene^26^,^27^. In this study, however, we established the HBV103-AdV system, and observed that the replicating HBV genome was abundantly produced *in vitro*. The application of CMV promoter and a foreign poly(A) signal in our construct resulted in AdV-mediated HBV replication being improved.

Even some AdVs, which couldn’t be produced by the conventional methods, have become readily available by using the improved preparation method of AdVs^28^. However, HBV103-AdVs were difficult to generate: 10 μg AdV genomes excised from cosmids generally induce full cytopathic effect (CPE) in almost all wells of a 96-well plate during vector proliferation in 293 cells, whereas those of HBV103-AdV showed CPE only in one-quarter to one-third of wells. The problem with HBV103-AdV productions may be the cause of the AdVs containing the CMV-pg RNA expression unit not being reported so far, although CMV promoter has frequently been used for expression of pg RNA in plasmid transfection. It is still unclear why the production of HBV103-AdV is difficult, but we experienced very similar phenomenon for Cre gene.

Adeno-associated virus (AAV) vectors appear to contrast remarkably with AdVs. AAV vectors are particularly useful for long-term experiments *in vivo* because of low immune responses. AAV expression lasts for several months or more, and hence the vectors containing the full-length HBV genome can develop hepatocellular carcinoma^29^ and fibrosis^30^ and offer superior animal models. In contrast, AdVs are used only for short-term experiments in *in vitro* and *in vivo*, although a certain type of AdV hardly induces immune responses and offers long-term expression^31^,^32^. Advantages of AdVs are that they highly and immediately express the purpose gene, foreign promoters and poly(A) signals can be utilized, and they can be used for various human hepatic cell lines including primary hepatocytes. Therefore, it seems important to choose the vectors appropriately to the purpose of the study.

Herein we demonstrated that the HBV103-AdV system induced much more efficient replication of the HBV genome than plasmid transfection: the replicating HBV genomes were easily and precisely detected from total cellular DNA using Southern blot analysis with no concentration step. Moreover, because AdVs can efficiently transduce PXB cells, the copy number of HBV genome was easily estimated as in HepG2 cells. Much higher transduction efficiencies were observed with AdVs than with plasmids, especially in the PXB cells, which are derived from the liver of humanized mice. Human primary hepatocytes derived from biopsy samples are hardly available because of ethical implications. Therefore the application of PXB cells to the HBV studies could become an alternative to reproduce natural HBV chronic infection *in vitro*. However, PXB cells have been limited to analysis using the HBV infectious particle, because plasmid transfection of PXB cells is extremely inefficient. Consequently, the HBV103-AdVs are likely to prove valuable for inducing efficient replication of the HBV genome in PXB cells. Additionally, mutagenesis analysis of the HBV genome can also be performed in PXB cells.

Homologous recombination probably occurs between the repeated sequences within the single AdV genome, while AdVs proliferate to more than 10^4^ copies in a vector-producing 293 cell. In this system, the pg RNA expression unit on the AdV consists of the minimal functional region for HBV genome replication, CMV promoter up to the 5′ cap site and the rabbit β-globin poly(A) sequence (Supplementary Fig. S3). When the 1.03 copy HBV genome, which possesses only 102 bp of overlapping sequence, was used for the transcription of pg RNA, the pseudo-ccc DNA was hardly observed. Although pseudo-ccc DNA may also be produced during plasmid proliferation, treatment with *Dpn*I, which digests only the plasmid-derived DNA molecules methylated in *E. coli*, probably excludes any pseudo-ccc DNA from detection of the replicating HBV genome. Additionally, pseudo-ccc DNA does not be detected using cell strains harboring multiple copies of HBV genomes, because those genomes are stable in the cell chromosomes.

Pseudo-ccc DNA should be identical to ccc DNA genome, which is the resultant genome of HBV proliferation, so the generation of pseudo-ccc DNA may hamper the accurate detection of the genuine ccc DNA genome. Because ccc DNA formation is essential to establishment of chronic infection of HBV, the precise detection and analysis of the ccc DNA genome is very important in HBV research.

The compact HBV103 genome, only 3.3 kb in length, might be applicable for various expression systems. If shorter promoter and poly(A) signal, e.g. small CB promoter and SV40 poly(A) signal, are employed for the HBV103 expression unit, the AAV containing the expression unit, which efficiently produces the pg RNA, could be constructed and used in *in vivo* study.

One of the most promising applications of the HBV103-AdV system is the screening of anti-HBV candidate drugs. Conventional drug screening for anti-HBV compounds in this field has been carried out, using either cell lines containing integrated HBV genomes or transfection with plasmids possessing HBV genomes. When using the HBV-producing cell lines, it is possible to quantify the precise number of copies of replicating HBV genome. However, the HBV-producing cells are derived from certain cell lines only, and the HBV multiplication steps cannot be controlled. In contrast, synchronous replication of the HBV genome can be carried out using plasmid transfection. Meanwhile, transfection efficiencies using plasmids do not reach 100% and the copy number of HBV genome inside cells varies greatly, so the replication efficiencies of the HBV genome differ between individual cells. In contrast, AdVs are able to transduce genomes evenly and efficiently to cells^33^, so by using HBV103-AdVs, the replication efficiency of the HBV genome is appropriately regulated in cells. Therefore, the HBV103-AdV system would provide a preferable screening method for estimating HBV genome replication.

We employed the S protein-deficient mutant “kS” and “ΔpreS” in the HBV103-AdV system, because the lack of S protein expression has been reported to boost virus DNA synthesis in the cells^34^. This elevated DNA synthesis, CMV promoter-driven pg RNA production and β-globin poly(A)-mediated stabilization of pg RNA, probably contributed to the increase of viral DNA accumulation in this system. By these reasons, we were able to shorten the experimental period to only 4 days, to detect replicating HBV genome with abundant signal far above background levels. In fact, using the microplate-based HBV103-AdV system, we showed that the respective EC_50_ values of the reverse transcriptase inhibitors, entecavir and lamivudine, were comparable to previously reported values obtained by both duck HBV infection of primary duck hepatocytes^23^ and plasmid transfection of human HBV^24^,^25^. As a result, it became clear that our system could accurately estimate the inhibitory potency of test compounds in drug screening. Moreover, since infectious particles are not released from the cells to the culture medium due to the defect in the S gene, the HBV103-AdVs can be safely handled.

One may want to construct the HBV103-AdV which produces wild-type HBV pg RNA (may be called CMwt AdV) based on the information of this work. However, it must be cautioned that CMwt AdV might cause acute hepatitis to the researchers who use this vector for the HBV study, because CMwt AdV efficiently produces pg RNA. It is advisable that the researchers should be vaccinated against HBV, at least, before generating CMwt AdV, and special care must be taken to use it in *in vivo* experiments. In contrast, AxCM103G-ΔpreS and AxCM103G-kS described here are considered safe because of non-infectivity of HBV. AxCM103G-ΔpreS, irreversibly deleted from a part of S gene, may be safer and is probably more suitable for drug screening.

In conclusion, the HBV103-AdV system, which efficiently transduced the HBV genome into both hepatoblastoma and primary cells using AdVs, is probably valuable to obtain quantitative results with high precision in HBV genome replication studies. Furthermore, investigation of HBV genome replication under various conditions can be performed using this system in a variety of cell types.

## Methods

### Cell culture, transfection, and AdV infection

Human 293^35^, Huh-7 and HepG2 cell lines are derived from the human embryonic kidney, human hepatocellular carcinoma and human hepatoblastoma, respectively. 293 cells were cultured in Dulbecco’s Modified Eagles Medium (DMEM) (Kohjin bio, Inc., Saitama, Japan) supplemented with 10% fetal calf serum (FCS). 293 cells constitutively express adenoviral E1 genes and support the replication of E1-substituted AdVs. Huh-7 and HepG2 cells were kept in high glucose DMEM supplemented with 10% FCS. PXB cells^7^,^8^ (PhoenixBio, Inc., Hiroshima, Japan), which are primary human hepatocytes, are derived from chimeric mice with humanized livers. PXB cells were cultured in hepatocyte clonal growth medium supplemented with 2% dimethyl sulfoxide (PhoenixBio, Inc., Hiroshima, Japan).

Lipofectamine^R^ LTX & PLUS^TM^ Reagent (Thermo Fisher Scientific, Inc., MA, USA) was used for plasmid transfection. The transfection was performed according to the manufacturer’s protocol (Thermo Fisher Scientific, Inc., MA, USA), and medium was exchanged after 24 h.

After infection with AdVs, the cells were maintained in DMEM supplemented with 5% FCS without geneticin.

### Plasmid and vector construction

Both the GFP-plasmid and the FG-AdV GFP-AdV possess a GFP expression unit, which consisted of CMV promoter^17^ and rabbit β-globin polyadenylation signal^18^.

All HBV genome expression units had been derived from pUC19 HBV C-JPNAT^36^, harboring genotype C (Accession No. AB246345). The mutant HBV genome ΔpreS lacks most of the preS region (nucleotide position 1040 to 1316 with position 1 corresponding to A nucleotide in the core gene initiation codon) and is replaced with a seven base-pair synthetic DNA, maintaining the reading frame. The deleted region and its structure exactly corresponds to HBV δ1^21^ of genotype A, while genotype C was used in this work. The nucleotide sequences are shown in Supplementary Fig. S4 ΔpreS.

The following mutations were introduced into the HBV genome, yielding mutant genome kS: T1471C, T1693C, which knocked out the initiation codon and the in-frame second ATG codon in the S gene, respectively. Because this second ATG corresponds to the initiation codon of genotype A, knockout of the first ATG of genotype C might still express functional S protein. Therefore, we knocked out both the first and second ATGs. C1486A and G1696C introduced stop codons in the S gene; C1462T and C1465A created a *BsrG*I site; A1684G and T1685A generated an *Aat*II site (Supplementary Fig. S4 kS).

The mutant HBV genome dP was obtained by deletion of the region between *Xba*I (1560 nt) and *Xcm*I (2009 nt) of the HBV genome (Supplementary Fig. S4 dP).

The mutant HBV expression units, under the control of the endogenous promoter and native poly(A) signal, started at 2728 nt and ended at 292 nt. Otherwise, the 5′-noncoding sequence of CMV promoter (−735 to +3 position from the 5′end of the cap nucleotide of CMV transcription) was connected to the HBV original 5′ cap nucleotide of pg RNA (3132 nt). This structure was designed so that the pg RNA of its native form was produced under the control of the CMV promoter. The original poly(A) signal (TATAAA, 16 to 21 nt) was disrupted and replaced by TATTATAA (*Psi*I cleavage sequence is underlined), and then connected with 460 bp DNA (*Bgl*II-*Pst*I) containing a poly(A) signal derived from the rabbit β-globin poly(A) fragment of pCAGGS^18^ (Supplementary Fig. S3).

### Vector generation and titration

All the AdVs described here were constructed using the cosmid cassette pAxcwit2 or pAxdV-FVF-4c containing the full-length AdV genome^28^,^37^. Purified and concentrated viral stocks were prepared as described in Kanegae *et al*.^38^. We experienced that HBV103-AdVs were not easily generated. One possible interpretation is as follows. At the initial step of AdV generation just after the transfection of the AdV genome to 293 cells, copy numbers of AdVs are very few. If the AdV expresses some protein or enzyme that decreases the replication of vector DNA genome, the cellular antiviral-defense mechanism may eliminate the vector genome. Nevertheless, in a small fraction of the cells AdV successfully escapes and replicates before the cellular antiviral mechanism is activated. Consequently, these cells produce many AdV particles, and then surrounding cells receive many more AdV particles. After several amplification cycles, a single AdV-infected cell produces many copies and a very high titer stock of AdVs is finally obtained. This hypothesis explains why the initial step of AdV generation is critical , and why these vectors, once obtained, can successfully be amplified.

AdVs were titrated using the method described by Pei *et al*.^39^. Briefly, copy numbers of viral genome that was successfully transduced into the infected target cells were measured by qPCR (relative virus titer: rVT). The rVT (copies/ml) normally corresponds to about one-fifth of the 50% tissue culture infectious dose (TCID_50_) titer or plaque assay. The sequences of the TaqMan probes for the titration are derived from the Ad5 pIX gene (Supplementary Table S1).

In *in vitro* experiments using AdVs, cytotoxicity and the titration methods sometimes influence the final results. Infection of AdVs at very high MOIs causes, in general, direct toxicity to cells. For example, although HepG2 cells infected with GFP-expressing AdVs at MOI 10 showed high level of expression (Fig. 1b, left, MOI10, and 1c, upper, MOI 10), cytotoxicity was observed at much higher MOIs. Therefore, AdVs must be used within the range lower than the cytotoxic level. Also, the differences in titration methods may influence the interpretation of the experimental results. The apparent titers of AdVs expressing a protein, Cre for example, which decreases growth of 293 cells, are often underestimated, and thus tend to cause over-infection and cytotoxicity. By using this titeration the over-and under-estimation of the AdV influencing the growth of 293 cells can be avoided.

### Conventional PCR

The PCR experiments were described previously^40^. Briefly, HepG2 cells in a 24-well plate were infected at MOI 1, 3 and 10, and incubated for 6 days postinfection. Total cellular DNAs were prepared and amplified by PCR with Tks Gflex DNA polymerase (Takara Bio, Inc., Shiga, Japan) and a PCR system (ProFlex^TM^ PCR system, Thermo Fisher Scientific, Inc., MA, USA). The PCR cycling conditions were follows: 94 °C for 1 min, followed by 25 cycles at 98 °C for 10 sec, 60 °C for 15 sec, and 68 °C for 30 sec. The sequences of the primers are described in Supplementary Table S2.

### Quantitative real-time PCR

HepG2 cells were transfected with AdVs and 3 days later the total RNAs of the infected cells were extracted using a DNA/RNA preparation kit (NucleoSpin, Macherey-Nagel, through Takara Bio, Inc., Shiga, Japan). Subsequently, the amounts of expressed mRNAs and human GAPDH (correction standard: Pre-Developed Assay Reagents Human GAPDH, Thermo Fisher Scientific, Inc., MA, USA) were quantified using reverse transcription (TaqMan Reverse Transcription Reagents, Thermo Fisher Scientific, Inc., MA, USA) and qPCR (Applied Biosystems ViiA7 real-time PCR system, Thermo Fisher Scientific, Inc., MA, USA); the ratio of pg RNA to GAPDH was then calculated. The sequences of the pg RNA primers were described in Supplementary Table S1.

The replicating HBV circular genome was quantified by qPCR analysis of infected total cellular DNA from cells that were incubated for 6 days postinfection. The sequences of the primers are also described in Supplementary Table S1.

### Measurement of expressed GFP

HepG2 and PXB cells were infected with each vector at MOI 1, 3 or 10 in a 24-well plate in triplicate experiments. Three days postinfection, the infected cells were washed twice with PBS(−). The cells were then fixed with 4% paraformaldehyde to quantify GFP fluorescence using Varioskan Flash (Thermo Fisher Scientific, Inc., MA, USA) or fluorescence microscopy (OLYMPUS IX71).

### Southern blot assay

HepG2 cells in a 6 well plate were infected with AdVs. At 6 days postinfection, the total cellular DNA was extracted and prepared as previously described^41^,^42^. Briefly, the cells were suspended in 0.7 ml of TNE-PK [50 mM Tris-HCl (pH 8.0), 100 mM NaCl, 10 mM EDTA, 100 mg/ml ptoteinase K], followed by the addition of SDS (0.1% final concentration). After incubation at 50 °C for 2 h, the mixture was extracted twice with phenol and twice with chloroform, and precipitated with twice volumes of ethanol at −20 °C for 1 h before washing once with 70% ethanol. The pellet was dissolved with TE containing 20 mg/ml RNase A.

Twenty μg of DNA from the AdV-infected cells were digested with 100 U *Kpn*I (Takara Bio, Inc., Shiga, Japan) at 37 °C for 4 h, while DNA from plasmid-transfected cells was treated with both 100 U *Hind*III (New England Biolabs, Inc., MA, USA) and 100 U *Dpn*I (New England Biolabs, Inc., MA, USA) at 37 °C for 4 h. Southern blot analysis was then perfomed as described in Maekawa *et al*.^37^.

### The HBV103-AdV system in microplate format

Huh-7 cells were infected with Ax-CM103G-ΔpreS or Ax-CM103G-dP at MOI 10, and seeded in a 96-well microplate at 3 × 10^4^ cells per well in 100 μl of high-glucose DMEM supplemented with 5% FCS. After incubation at 37 °C for overnight, 2 μl of the reverse transcriptase inhibitors, entecavir (Toronto Research Chemicals, Inc., Toronto, Canada) and lamivudine (Wako Pure Chemical Industries, Ltd., Osaka, Japan), were added at various concentrations (0.1% DMSO final concentration), and the plate was maintained for further 3 days.

The cells were then washed with PBS(−), and total DNA was extracted by incubation at 50 °C for 2 h in 100 μl of DNA extraction buffer [50 mM Tris-HCl (pH 8.5), 16.6 mM ammonium sulfate, 5 mM 2-mercaptoethanol, 5 mM MgCl_2_, 0.01% SDS, 5 μg of proteinase K], followed by heat inactivation at 95 °C for 30 min. HBV rc DNA and ccc DNA were quantified by qPCR with Universal SYBR Select Master Mix (Thermo Fisher Scientific, Inc., MA, USA) and the following primers: 5′-GTTGGGGGAGGAGATTAGGT-3′ and 5′-GCTTGCCTGAGTGCTGTATG-3′.

The 50% effective concentration (EC_50_) was calculated by four-parameter logistic curve fitting.

Coefficient of variation, signal-to-background ratio, and signal-to-noise ratio in the assay were calculated as CV = σs/μs × 100, S/B = μs/μb, and S/N = (μs − μb)/σb, respectively, where μs and μb are the means of the signal (Ax-CM103G-ΔpreS infection) and background (Ax-CM103G-dP infection), respectively, and σs and σb are the standard deviations of the signal and background, respectively. The Z’ factor was calculated according to the equation Z’ = 1 − (3σs + 3σb)/(μs − μb), where μs, μb, σs, and σb have been defined above^22^.

## Additional Information

**How to cite this article**: Suzuki, M. *et al*. Efficient genome replication of hepatitis B virus using adenovirus vector: a compact pregenomic RNA-expression unit. *Sci. Rep.* **7**, 41851; doi: 10.1038/srep41851 (2017).

**Publisher's note:** Springer Nature remains neutral with regard to jurisdictional claims in published maps and institutional affiliations.

## Supplementary Material

Supplementary Information
