# Supplementary material for: Efficient genome replication of hepatitis B virus using adenovirus vector: a compact pregenomic RNA-expression unit
Source: Sci Rep. 2017 Feb 3;7:41851. doi: 10.1038/srep41851 (PMC5291108; doi:10.1038/srep41851)
Supplement: Supplementary Information [file srep41851-s1.pdf]

Efficient genome replication of hepatitis B virus using adenovirus vector: a compact pregenomic RNA-expression unit

Mariko Suzuki[1], Saki Kondo[1], Manabu Yamasaki[2], Norie Matsuda[2], Akio Nomoto[2†], Tetsuro Suzuki[3], Izumu Saito[1*], Yumi Kanegae[1,4]

1, Laboratory of Molecular Genetics, The Institute of Medical Science, The University of Tokyo, Minato-ku, Tokyo, Japan
2, Laboratory of Virology, Institute of Microbial Chemistry (BIKAKEN), Microbial Chemistry Research Foundation
3, Department of Virology and Parasitology, Hamamatsu University School of Medicine
4, Core Research Facilities of Basic Science (Molecular Genetics), Research Center for Medical Science, Jikei University School of Medicine
†, Deceased

Correspondence should be addressed to I.S (isaito@ims.u-tokyo.ac.jp).
Address and phone: 4-6-1 Shirokanedai, Minato-ku, Tokyo, Japan, 108-8639
+81-3-5449-5556 (phone), +81-3-5449-5432 (Fax)

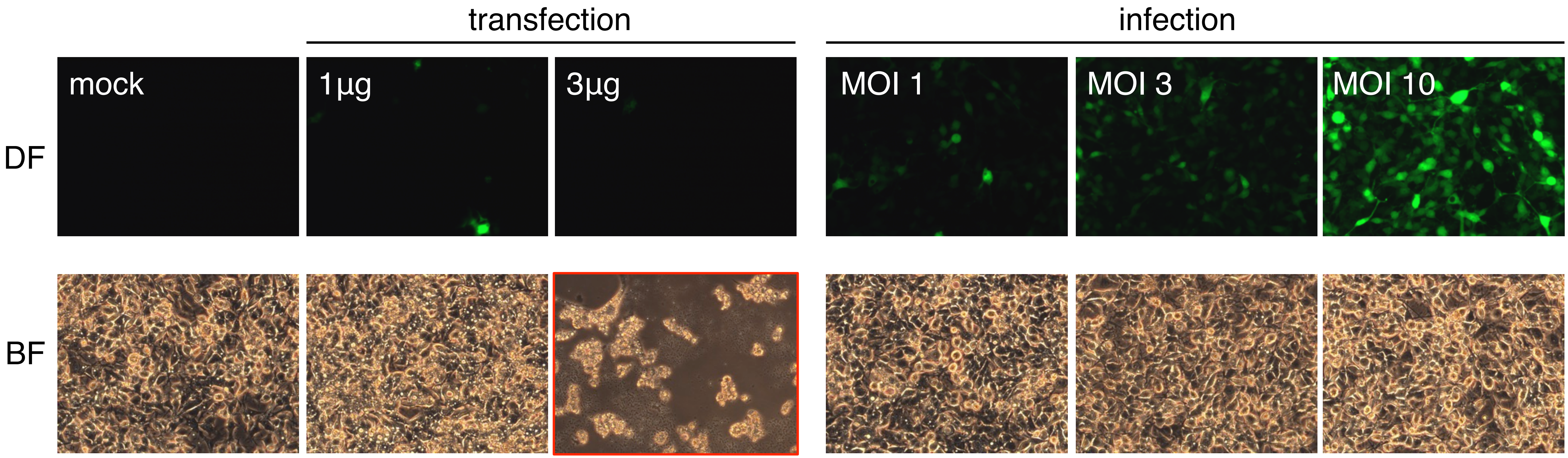

Supplementary Figure S1. Optimisation of the plasmid transfection experiment in HepG2 cells. (a) Images of GFP fluorescence was observed using microscopy. Cells were transfected with GFP-expressing plasmids or infected with AdVs expressing GFP at the indicated amount and MOIs. *n*=3. Mock, mock infection of the indicated cells; DF, dark-field images; BF, bright field images.

a

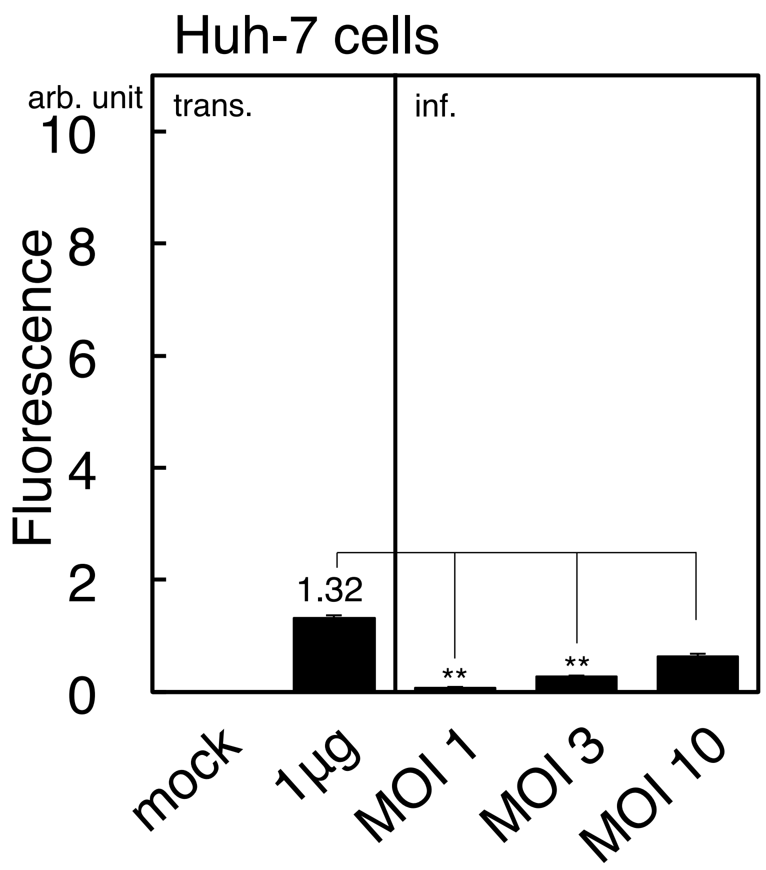

b

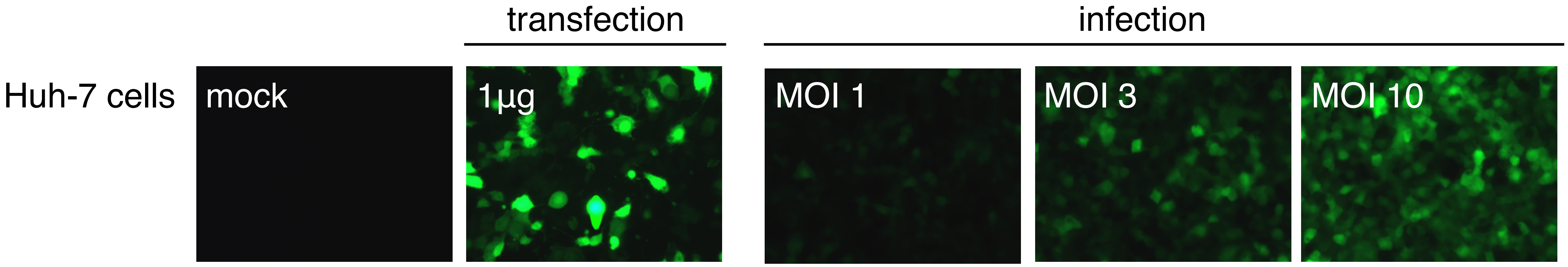

Supplementary Figure S2. Transduction efficiencies of plasmids and AdVs in Huh-7 cells. (a) GFP fluorescence was quantified using Varioskan Flash. Cells were transfected with GFP-expressing plasmids or infected with AdVs expressing GFP at the indicated amount and MOIs. 'arb. unit', arbitrary unit. *n*=3. (b) Images obtained using fluorescence microscopy in the same manner as that described in (a). Error bars represent ±s.d.; mock, mock infection of the indicated cells; **P<0.01.

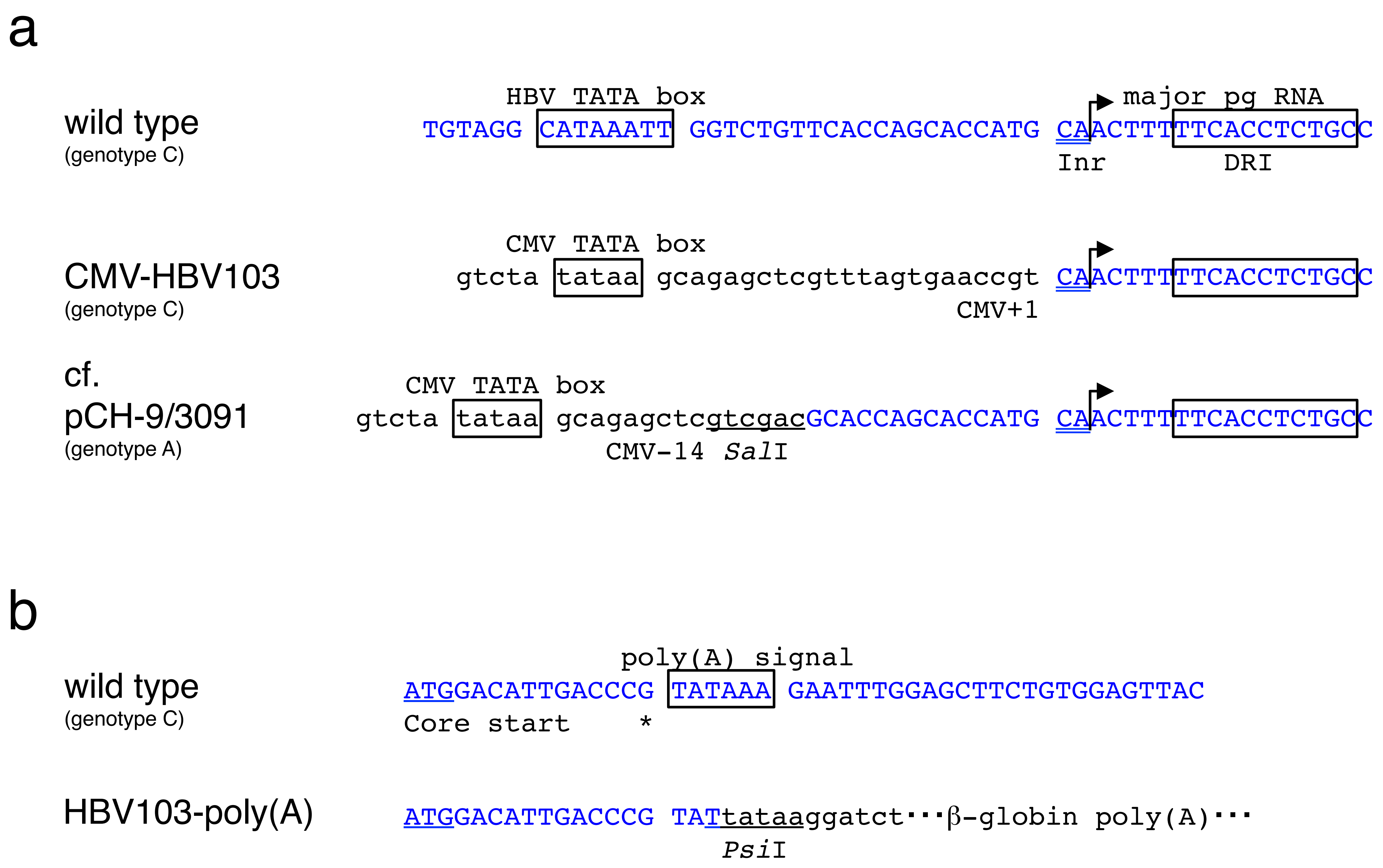

Supplementary Figure S3. Nucleotide sequences of pg RNA expression units. Capital letters and small letters represent HBV genome and foreign sequences, respectively. (a) Junction sequences between the promoter and HBV genome. (b) Junction sequences between the HBV genome and poly(A) signal. Arrows, major initiation site of transcription; Inr, transcription initiator (CA dinucleotides); DRI, direct repeat 1; underlined, recognition sequence of the restriction enzyme; asterisk, genotypic variation (T in genotypes A and B).

ΔpreS

```
S frame                                                1039          deletion from 1040 nt to 1316 nt
1001   T CCC AAT CCT CTG GGA TTC TTT CCC GAT CAC CAG TTG GAC CCG GCG TTC GGA GCC AAC TCA AAC AAT CCA GAT TGG GAC TTC AAC CCC AAC AAG GAT CAT   1100

1101   TGG CCA GAG GCA AAT CAG GTA GGA GCG GGA GCA TTC GGG CCA GGG TTC ACC CCA CCA CAC GGC GGT CTT TTG GGG TGG AGC CCA CAG GCA CAG GGC GTA T   1200

1201   TG ACA ACC GTG CCA GTA GCA CCT CCT CCT GCC TCC ACC AAT CGG CAG TCA GGA AGA CAG CCT ACT CCC ATC TCT CCA CCT CTA AGA GAC AGT CAT CCT CA   1300
                                          1317
1301   G GCC ATG CAA TGG AAC t ccg aat TCC ACA ACA TTC CAC CAA GCT CTG CTA GAC CCC AGA GTG AGG GGC CTA TAT CTT CCT GCT GGT GGC TCC AGT TCC G   1400
             Met (preS2)      synthetic DNA carrying EcoRI site
```

kS

```
S frame
1401   AAC CCT GTT CCG ACT ACT GCC TCA CCC ATA TCG TCA ATC TTC TCG AGG ACT GGG GAC CCT GTA CAG AAC ACG GAG AAC ACA ACA TAA GGA TTC CTA GGA C   1500
                                                                                 BsrGI     1st ΔATG of SS protein   *** stop codon
1501   CC CTG CTC GTG TTA CAG GCG GGG TTT TTC TTG TTG ACA AGA ATC CTC ACA ATA CCA CAG AGT CTA GAC TCG TGG TGG ACT TCT CTC AAT TTT CTA GGG GG   1600

1601   A GCA CCC ACG TGT CCT GGC CAA AAT TCG CAG TCC CCA ACC TCC AAT CAC TCA CCA ACC TCT TGT CCT CCA ATT TGT CCT GGC TGA GTC TGG ACG TCT CTG   1700
                                                                                             stop codon ***     AatII, 2nd ΔATG
1701   CGG CGT TTT ATC ATA TTC CTC TTC ATC CTG CTG CTA TGC CTC ATC TTC TTG TTG GTT CTT CTG GAC TAC CAA GGT ATG TTG CCC GTT TGT CCT CTA CTT C   1800
```

dP

```
Pol frame    ▶Pol RT region (1505 nt to 2476 nt)                            1559         deletion from 1560 nt to 2009 nt
1501   C CCT GCT CGT GTT ACA GGC GGG GTT TTT CTT GTT GAC AAG AAT CCT CAC AAT ACC ACA GAG TCT AGA CTC GTG GTG GAC TTC TCT CAA TTT TCT AGG GGG   1600
                                                                                 XbaI
1601   AGC ACC CAC GTG TCC TGG CCA AAA TTC GCA GTC CCC AAC CTC CAA TCA CTC ACC AAC CTC TTG TCC TCC AAT TTG TCC TGG CTA TGT CTG GAT GTG TCT G   1700

1701   CG GCG TTT TAT CAT ATT CCT CTT CAT CCT GCT GCT ATG CCT CAT CTT CTT GTT GGT TCT TCT GGA CTA CCA AGG TAT GTT GCC CGT TTG TCC TCT ACT TC   1800

1801   C AGG AAC ATC AAC TAC CAG CAC GGG ACC ATG CAA GAC CTG CAC GAT TCC TGC TCA AGG AAC CTC TAT GTT TCC CTC TTG TTG CTG TAC AAA ACC TTC GGA   1900

1901   CGG AAA CTG CAC TTG TAT TCC CAT CCC ATC ATC CTG GGC TTT CGC AAG ATT CCT ATG GGA GTG GGC CTC AGT CCG TTT CTC CTG GCT CAG TTT ACT AGT G   2000
                 2010
2001   CC ATT TGT TCA GTG GTT CGT AGG GCT TTC CCC CAC TGT TTG GCT TTC AGT TAT ATG GAT GAT GTG GTA TTG GGG GCC AAG TCT GTA CAA CAT CTT GAG TC   2100
                 XcmI
```

Supplementary Figure S4. Nucleotide sequences of mutant HBV genomes. Nucleotide position 1 corresponds to A nucleotide in the core gene initiation codon. The mutant HBV genome ΔpreS lacks most of the preS region and was replaced with a seven base-pair synthetic DNA, maintaining the reading frame. The following mutations were introduced into the HBV genome, yielding the mutant genome kS: T1471C, T1693C, knocked out the initiation codon and the in-frame second ATG codon in the SS gene, respectively; C1486A, G1696C, introduced stop codons in the SS gene; C1462T, C1465A, created a *BsrG*I site; A1684G, T1685A, generated an *Aat*II site. The mutant HBV genome dP was obtained by deletion of the region between *Xba*I (1560 nt) and *Xcm*I (2009 nt) of the HBV genome.

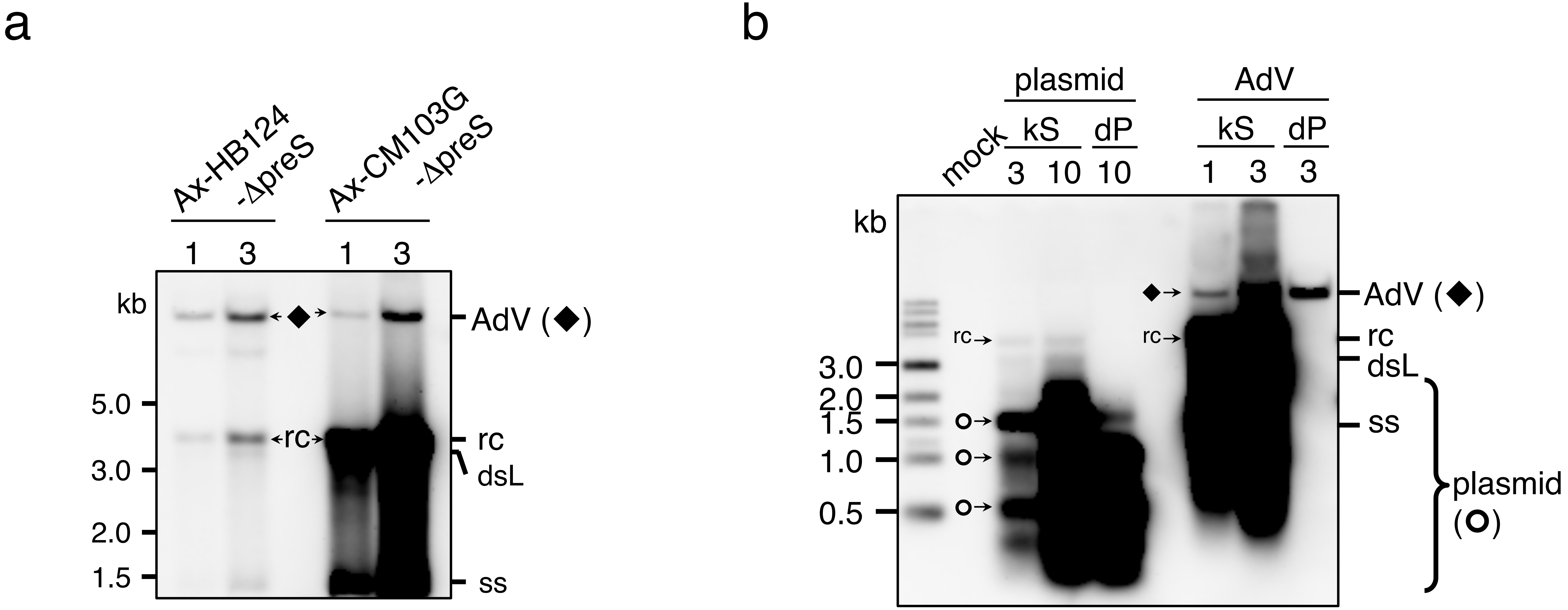

Supplementary Figure S5. Overexposure of Southern blot. Over-exposition of the blot was necessary to reveal the low signals. (a) Overexposure of Figure 2c. HepG2 cells were infected with Ax-HB124-ΔpreS and Ax-CM103G-ΔpreSat the indicated MOIs. The representations are the same as in Figure 2c. (b) Overexposure of Figure 4a. HepG2 cells were infected with Ax-CM103G-kS (kS) or Ax-CM103G-dP (dP) at the indicated MOIs, or transfected with plasmids possessing the same mutant HBV expression units. The representations are the same as in Figure 4a. Full-length blots are presented in Supplementary Figure S9.

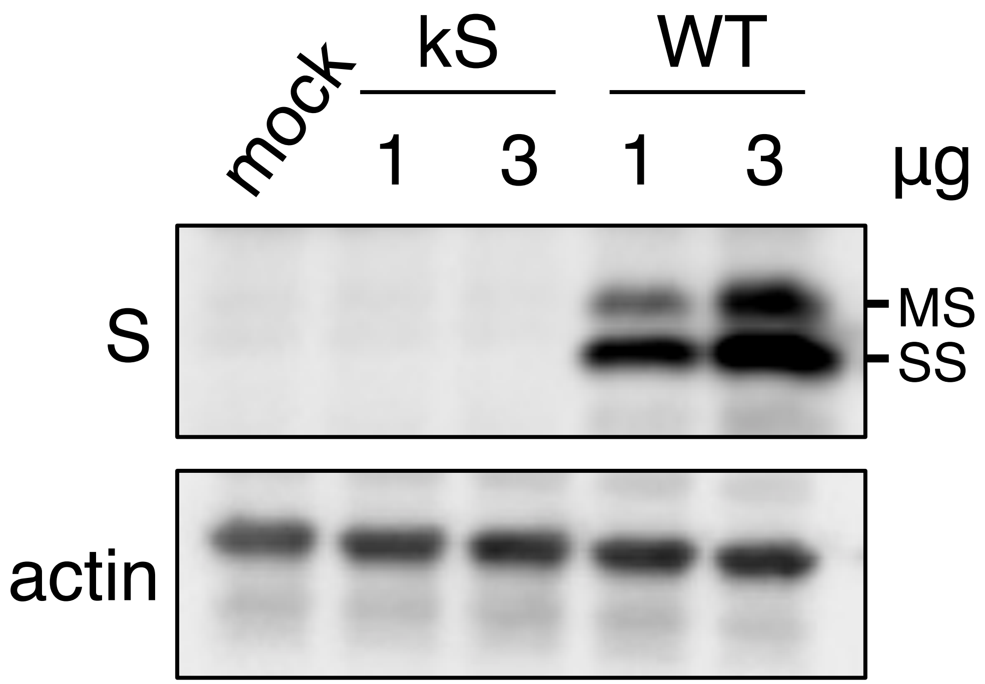

Supplementary Figure S6. MS and SS proteins are not detected in Huh-7 cells transfected with kS-expressing unit. Total protein was extracted from transfected cells, and S protein (top) and actin (bottom) were detected by western blot analysis. kS, plasmid possessing the mutant HBV genome kS; WT, plasmid possessing wild type HBV genome.
Two days after transfection, Huh-7 cells were harvested and total protein was extracted using NP-40 lysis buffer containing 50mM Tris-HCl (pH 8.0), 0.15M NaCl, 5mM EDTA, 1% NP-40. The lysates were mixed well in a rotator for 2 h at 4°C, centrifuged at 15,000 rpm for 5 min at 4°C, and the supernatants were collected. Western blotting was performed as described previously[43]. The membrane was incubated for 2 h at room temperature in the presence of anti-S monoclonal antibody (#2AHB16, Institute of Immunology Co., LTD, Tokyo, Japan) diluted to 0.3 µg/ml with PBS-Tween, followed by incubation with peroxidase-conjugated goat anti-mouse IgG+IgM (#115-035-068, Jackson Immunoresearch, PA, USA) diluted to 1/10,000 with PBS-Tween. An anti-actin peptide goat polyclonal antibody (#sc-1616, Santa Cruz Biotechnology, CA, USA) diuted to 1/200 was also detected to show equal loading.

43. Baba, Y., Nakano, M., Yamada, Y., Saito, I. & Kanegae Y. Practical Range of Effective Dose for Cre Recombinase-Expressing Recombinant Adenovirus without Cell Toxicity in Mammalian Cells. Microbiol Immunol 49, 559-570 (2005)

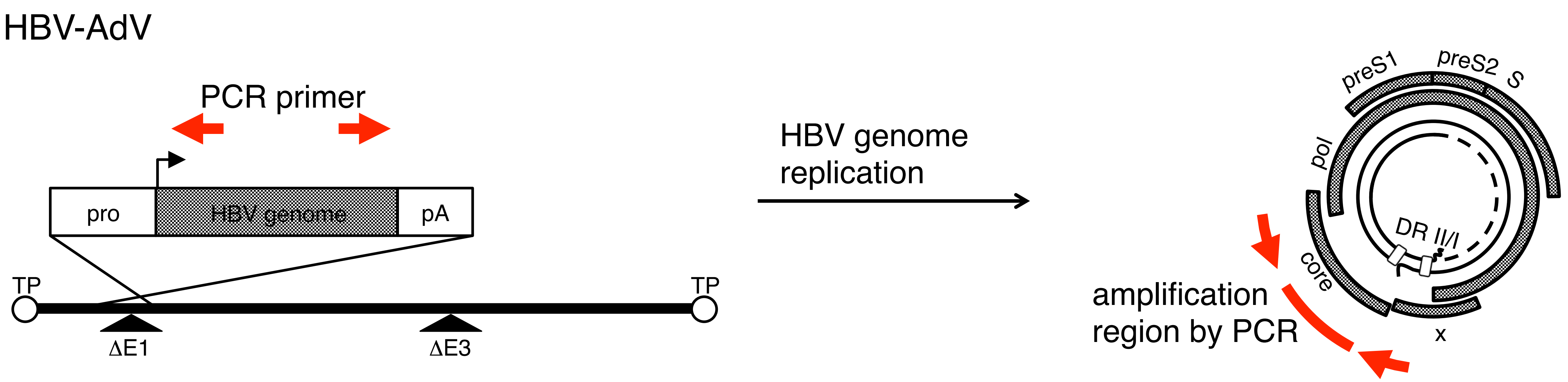

Supplementary Figure S7. Strategy for detection of circular HBV genome. Schematic represent the detection of circular HBV genome, which is the result of genome replication. The box containing ‘pro’, ‘HBV genome’ and ‘pA’ represents the expression unit and red arrows show the primer location for PCR amplification. Pro, CMV and endogenous promoter of HBV; pA, poly(A) signal; hatched box, HBV genome/coding region; black bold line, AdV genome; TP, terminal protein; ΔE1, E1 cloning site; ΔE3, E3 deletion region.

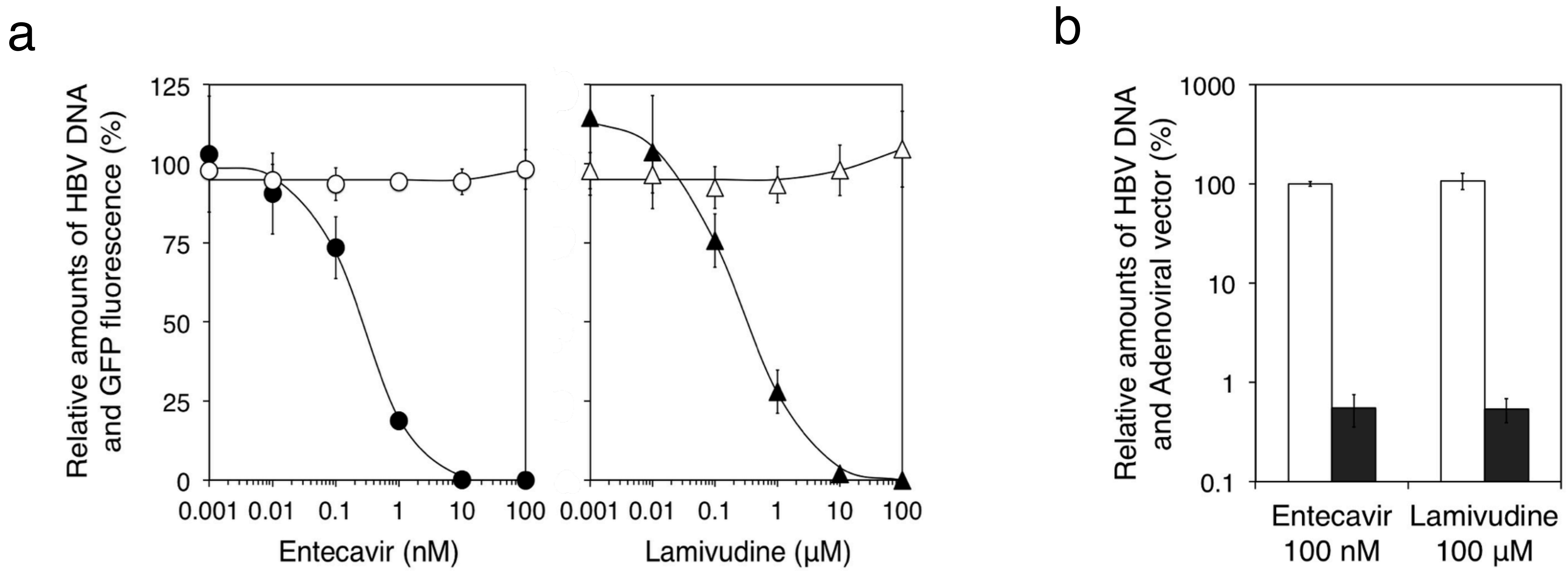

Supplementary figure S8. Effects of HBV reverse transcriptase inhibitors on GFP fluorescence. (a) and adenoviral vector (b) in Huh-7 cells coinfected with HBV103-AdV and GFP-AdV. Huh-7 cells were coinfected with Ax-CM103G-ΔpreS (MOI 5), and GFP-AdV (MOI 5) and incubated with entecavir and lamivudine. GFP fluorescence at 4 days postinfection was quantified by Varioskan Flash (opened symbols in panel a). Replicating HBV genomes (closed symbols in panel a and black bars in panel b) and adenoviral vectors (white bars in panel b) in cells were quantified by qPCR. Relative amounts of the samples are shown as a percentage of the untreated control (DMSO). *n*=3. Error bars represent ±s.d.

Supplementary Table S1 | **Primers for quantitative real-time PCR**

purpose	target	orientation	sequence (5'-3')
vector titration	pIX	Forward	TGTGATGGGCTCCAGCATT
		Reverse	TCGTAGGTCAAGGTAGTAGAGTTTGC
		Probe	ATGGTCGCCCCGTCCTGCC
	Actin	Forward	CTCGCAGCTCACCATGGAT
		Reverse	ATGCCGGAGCCGTTGTC
		Probe	ATGATATCGCCGCGCTCGTCGT
qPCR for core/pg RNA	Core	Forward	GCCTTCTGACTTCTTTCCTTCTATTC
		Reverse	GACTCTAAGGCCTCCCGATACA
		Probe	AGATCTCCTCGACACCGCCTCAGCT
qPCR for the replicating HBV genome	circular genome	Forward	CGGCACCGACAACTCTGTT
		Reverse	GCTGTATGGTGAGGAGAACAATGTT
		Probe	CACTTCGCTTCACCTCTGCAC

Supplementary Table S2 | **Primers for PCR**

target	orientation	sequence (5'-3')
Circular HBV genome	Forward	GCGCTTGAGGCATACTTCAAAGACTGTTTG
	Reverse	GCGGAACAGTTTCTCTTCCAAAAGTAAGAC
E4	Forward	CGCGGCAGCAGCGGATGATCCTCCAGTATG
	Reverse	GCGACTACTACACAGAGCGATCTAAGCGG
GAPDH	Forward	CGCGCTTAGC ACCCCTGGCCAAGGTCATCC
	Reverse	GCGTCTAGACGGCAGGTCAGGTCCACCAC

Fig. 2c

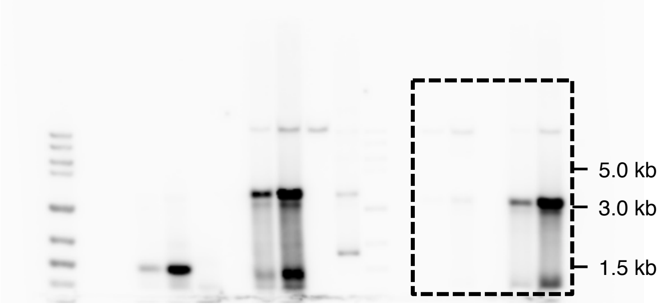

Fig. 3b

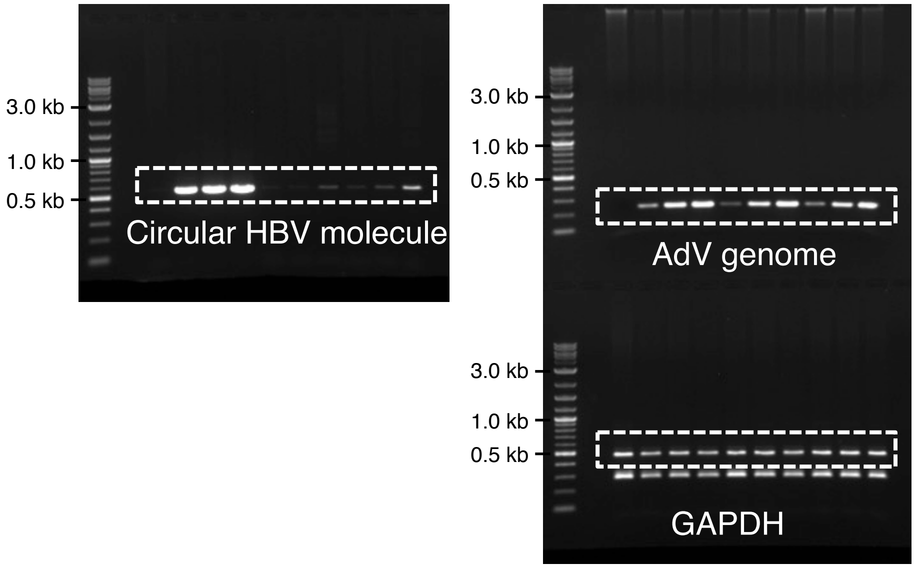

Fig. 4a

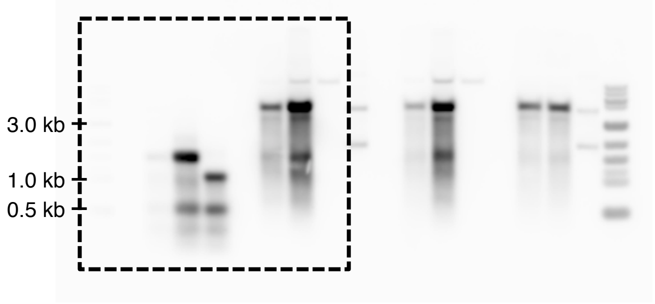

Supplementary Fig. S5

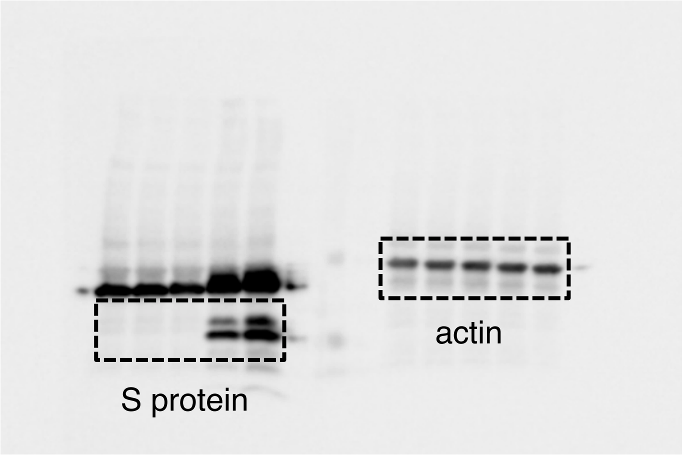

Supplementary Fig. S6a

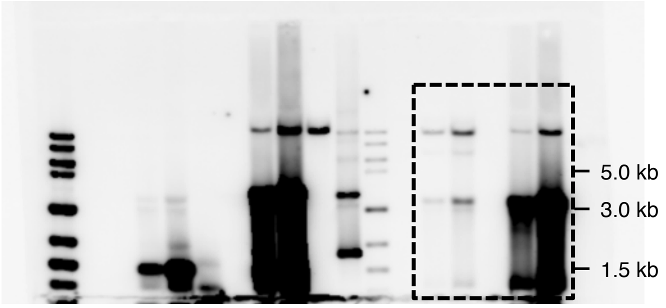

Supplementary Fig. S6b

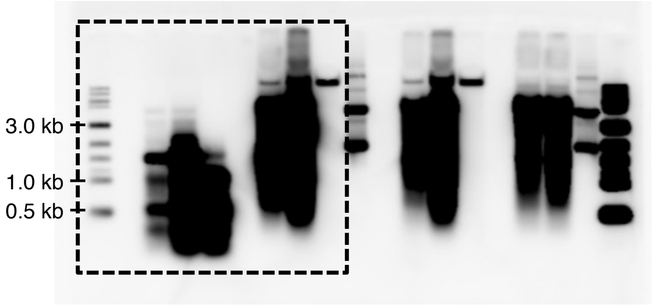

Supplementary Figure S9. Uncropped, unprocessed images.
